# Modelling the impact of population-based cytologic screening on cervical cancer incidence and mortality in Hong Kong: an age–period–cohort approach

**DOI:** 10.1038/sj.bjc.6602805

**Published:** 2005-10-04

**Authors:** P P S Woo, T Q Thach, S T B Choy, S M McGhee, G M Leung

**Affiliations:** 1 Department of Community Medicine and School of Public Health, University of Hong Kong, 21 Sassoon Road, Pokfulam, Hong Kong, China; 2 Department of Mathematical Sciences, University of Technology, Sydney, Australia; 3 Takemi Program, Harvard School of Public Health, 677 Huntington Avenue, Boston, MA 02115, USA

**Keywords:** cervical cancer, age–period–cohort, screening, maximum likelihood estimation, Bayesian methods, Hong Kong

## Abstract

Cervical cancer incidence and mortality statistics in Hong Kong during 1972–2001 were examined to estimate the potential number of cancer cases that can be averted and years of life saved after the launch of an organised, population-based cytologic screening recall programme in 2004 with projections to 2016. Incidence rates under the *status quo* of opportunistic screening were projected by an age–period–cohort model, using maximum likelihood and Bayesian methods. Modelled rates were translated into numbers of cancer cases and deaths using mid-year population figures and age–period-specific mortality to incidence ratios. We applied International Agency for Research on Cancer risk reduction estimates for different screening strategies to these base case figures to estimate the number of incident cancers potentially averted and years of life saved attributable to organised screening incremental to the current *status quo*. The estimated numbers of cases projected to be preventable by the maximum likelihood (Bayesian) approach from 2002 to 2016 were 4226 (4176), 3778 (3728) and 2334 (2287) with organised screening every 1, 3 and 5 years, compared to haphazard screening currently. Correspondingly, 33 000 (32 800), 29 500 (29 300) and 18 200 (17 900) years of life could potentially be saved.

Although the efficacy of cytologic screening had never been formally tested in randomised controlled trials, there is wide consensus based on historical data and observational epidemiology that screening leads to significant reductions in both invasive cancer incidence and mortality (Miller *et al*, 1976; La Vecchia *et al*, 1984; Laara *et al*, 1987; Eddy, 1990; Herrero *et al*, 1992; Quinn *et al*, 1999; Peto *et al*, 2004). Hong Kong first implemented population-based screening in March 2004 when the government launched its organised recall programme despite an appreciable cervical cancer burden (Leung *et al*, 2005), the availability of financial resources (annual GDP per capita in 2003=$22 991) and an otherwise adequate public health infrastructure to sustain such a programme (Department of Health, 2003). Opportunistic screening had been increasingly available since the late 1970s and early 1980s. By 2003, 42–60% (depending on the degree of potential under-reporting) of women aged 21 years or over had been screened at least once within the last 5 years (Leung *et al*, 2005).

To examine and quantify the potential public health impact of this new preventive programme relative to the *status quo* of haphazard screening, we adopted the age–period–cohort (APC) approach, which has been used extensively to predict future incidence and mortality trends under different public health intervention scenarios (Osmond, 1985; Dyba and Hakulinen, 2000; Møller *et al*, 2003; Quinn *et al*, 2003). Our primary objective was to project the number of incident cancers potentially averted and years of life saved (YLS) attributable to the new organised screening programme through 2016, by modelling historical incidence and mortality data from 1972 to 2001 using maximum likelihood and Bayesian methods.

## MATERIALS AND METHODS

### Sources of data

Data on cervical cancer incidence and mortality from January 1972 to December 2001 were based on records of the Hong Kong Cancer Registry. A total of 15 140 incident cases (out of a total of 15 238 incident cases where the age at diagnosis was unknown in 98 cases) and 4230 deaths of invasive cervical cancer were included in the present analysis. Statistics on actual and estimated (beyond 2004) mid-year population figures were obtained from the Census and Statistics Department.

Incidence data were grouped from 1972–76 to 1997–2001 into 5-year periods and 5-year age groups from 25–29 to 80–84 years, to give synthetic birth cohorts centred at 5-year intervals since 1892. Age groups below 25 and above 85 years were omitted because of small numbers.

### Age–period–cohort projection of *status quo* to 2016

#### Maximum likelihood approach

We modified a previously developed APC model (Leung *et al*, 2005) and fitted the data by Poisson regression to compute 15-year projections of incidence rates to the period 2012–16. Let *c*_*ij*_ be the observed cases for age group *i* in time period *j*. We assumed that it follows a Poisson distribution with mean *μ*_*ij*_, that is, *c*_*ij*_∼Poisson(*μ*_*ij*_), and we modelled the mean as 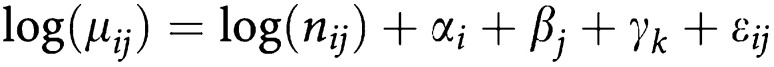 where *α*_*i*_ is the age effect (*i*=1, …, *I*), *β*_*j*_ is the period effect (*j*=1, …, *J*), *γ*_*k*_ is the cohort effect (*k*=1, …, *K* where *k*=*I*+*j*−*i* and *K*=*I*+*J*−1), *n*_*ij*_ denotes the total number of person-years for age group *i* in time period *j* and *ε*_*ij*_ is the random error term.

We applied linear extrapolation of the six observed periods and the six most recent birth cohorts based on data from 1972 to 2001 (Osmond, 1985; Negri *et al*, 1990; Bray, 2002). This set of projected rates would reflect a continuation of the *status quo* of opportunistic screening in Hong Kong through 2016 (base case). The autoregressive nature of our method assumes that current trends will continue in the future. However, this assumption may not hold if, say, the future rate of increase in screening uptake is higher (e.g. as a result of introduction of organised programme) or lower (e.g. as a result of saturation effect). Therefore, to quantify the sensitivity of our projection estimates about the continuation of current trends, we varied future period and cohort effects from −5 to +5% per 5-year time period over the base case period and cohort effects, following Osmond (1985). All computations were performed using SAS version 8.02.

#### Bayesian approach

For comparison purposes, we applied the Bayesian framework to the APC modelling. A second-order autoregressive model was specified to smooth the effects of age, period and cohort, thus guarding against excessive deviation of the parameter estimates from those in adjacent time bands. The degree of smoothing was learned from the data on each time scale. The expected value for each effect was then based on an extrapolation from its two immediate predecessors. For the age effects *α*_*i*_: 
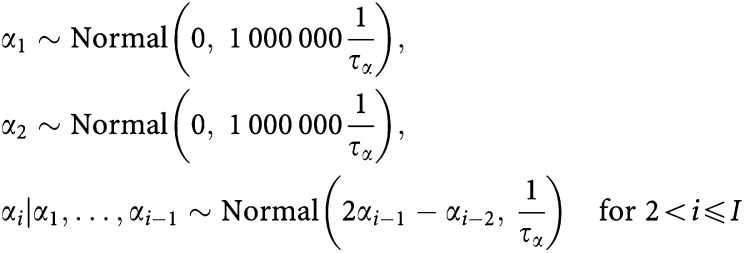
 where the hyperparameter *τ*_*α*_ was a precision parameter determining the smoothness of the age effect and was given a highly noninformative prior, namely, *τ*_*α*_∼Gamma(0.001, 0.001). The same type of prior was used for the period and cohort parameters *β*_*j*_ and *γ*_*k*_ with precision parameters *τ*_*α*_ and *τ*_*γ*_, respectively (Breslow and Clayton, 1993; Bashir and Esteve, 2001; Bray *et al*, 2001, 2002). Estimated future rates were computed by combining the estimates of the age, period and cohort effects obtained. Parameter estimates and 90% credible intervals were obtained by Markov Chain Monte Carlo (MCMC) methods. The simulations were run for 12 000 iterations with the first 2000 iterations used as burn-in to minimise the effect of initial values. As successively sampled values were dependent, samples at suitable spacings of 10 were picked off to mimic a random drawing of 1000 samples from the posterior. As with the maximum likelihood APC model, this set of Bayesian projected rates reflected an extension of the current situation of opportunistic screening continued to 2016. We also varied the future period and cohort effects from −5 to +5% per 5-year time period as we did for the maximum likelihood estimates. This model was implemented using BUGS (http://www.mrc-bsu.cam.ac.uk/bugs/welcome.shtml).

### Estimation of potential cases averted and years of life saved from new organised screening programme

We applied the projected future incidence rates, derived using maximum likelihood and Bayesian techniques as described above, to provisional figures of the mid-year female population for the respective years to calculate the expected number of new cancer cases through 2016. Population mortality rates were combined with incidence rates to derive age–period-specific mortality to incidence (*M*/*I*) ratios using observed data from 1972–76 to 1996–2001 (Taylor *et al*, 2001). Assuming no change in cancer-specific survival from potentially improved management of the disease over the projected time horizon, thereby isolating the effect of screening on incidence reduction, we applied a constant set of age–period-specific *M*/*I* ratios that were based on the two most recent observed periods and smoothed using moving averages. The numbers of cancer-specific deaths to 2016 under the *status quo* scenario of opportunistic screening were then calculated from the numbers of new cancer cases and the application of *M*/*I* ratios to incidence rates in the projection period.

To assess the impact of the new organised screening programme, we computed the number of cancer cases under different screening frequencies by applying the risk reduction estimates as per the International Agency for Research on Cancer (IARC). The IARC study, which comprised large screening programmes in eight European and North American centres for over 20 years (IARC Working Group, 1986), estimated that the percentage reductions in the cumulative incidence of cervical cancer in women aged 35–64 years were 91.6, 83.9, 54.0 with every 1-, 3- or 5-year organised screening, respectively, compared to opportunistic screening. We derived these figures by calibrating the original IARC estimates, which were based on the comparator scenario of no screening, to Hong Kong's *status quo* of opportunistic screening. Specifically, we calculated the expected incidence reduction according to the opportunistic screening pattern as described below, which yielded a 22.4% lower incidence compared with no screening. We then used this as the new baseline to which organised screening patterns were compared.

Based on the representative 2003 Population Health Survey (University of Hong Kong, 2005) and a subsequent published report in interpreting the data (Leung *et al*, 2005), the pattern of opportunistic screening in local women was estimated as follows: −40.7% had never been screened, 15.4% had at least one screen by age 30 years, 19.6% had at least one screen by age 50 years, 0.1% were screened regularly every 5 years, 0.5% every 4 years, 3.3% every 3 years, 4.9% every 2 years and 15.4% annually.

Projected incident case numbers obtained from the APC modelling were adjusted downwards based on these cancer incidence reduction figures, beginning from the period 2007–11, assuming that all Hong Kong women would derive a similar level of benefit from screening compared to populations in the IARC study and irrespective of age and other characteristics. We assumed that the full benefit of the organised screening programme would only begin from 2007. Estimates for the first projected period of 2002–06 were obtained by backward projecting the 2007 to 2016 figures, assuming a constant decrease in incidence over the six observed periods. The numbers of cancer-related deaths were then scaled *pro rata* according to the procedure using age–period-specific *M*/*I* ratios as specified above.

Lastly, we multiplied the number of deaths in each age group by the life expectancy at the mean age of death within each group based on the Hong Kong Life Table (Hong Kong Census and Statistics Department, 2004) to calculate the number of years of life lost (YLL) as a result of cervical cancer (Murray and Lopez, 1996). The marginal difference in YLL between the various screening scenarios yielded additional YLS attributable to each strategy benchmarked against the comparator of opportunistic screening.

## RESULTS

### Goodness-of-fit of maximum likelihood and Bayesian APC models

Table 1 shows the change in deviance, a measure of goodness-of-fit, in the sequential building of the maximum likelihood APC models. Both the age–period (AP) and age–cohort (AC) models significantly improved the fit over the age only and age–drift models. The full three-factor model was in turn significantly better than the two-factor AP (*P*<0.001) and AC (*P*<0.001) models by the F-test (McCullagh and Nelder, 1983), and was therefore adopted to project future incidence rates by linear extrapolation of both the period and cohort effects (Osmond, 1985).

The Bayesian APC model also achieved a good fit as indicated by convergence diagnostics and autocorrelation statistics. Figure A of the appendix (online) presents convergence diagnostic plots on selected age, period and cohort parameters from the Bayesian model. These particular parameters were chosen for illustrative purposes, as they captured the largest number of cancer cases in the 50–54 years age group, the birth cohort with central year of birth in 1942 and 2002–06 was the first projected period. (A full set of convergence diagnostic plots is available from the authors on request.) The time-series plots of the MCMC iterations demonstrate convergence of the Markov chains. Figure B of the appendix (online) shows autocorrelation plots up to lag 50 for selected parameters, chosen to represent different age groups (young, middle and old), years at diagnosis and birth cohorts equally spaced over the time horizon of the study period. Independence between samples is confirmed, as the autocorrelations are near zero for all time-lag separations.

**Figure 1:**
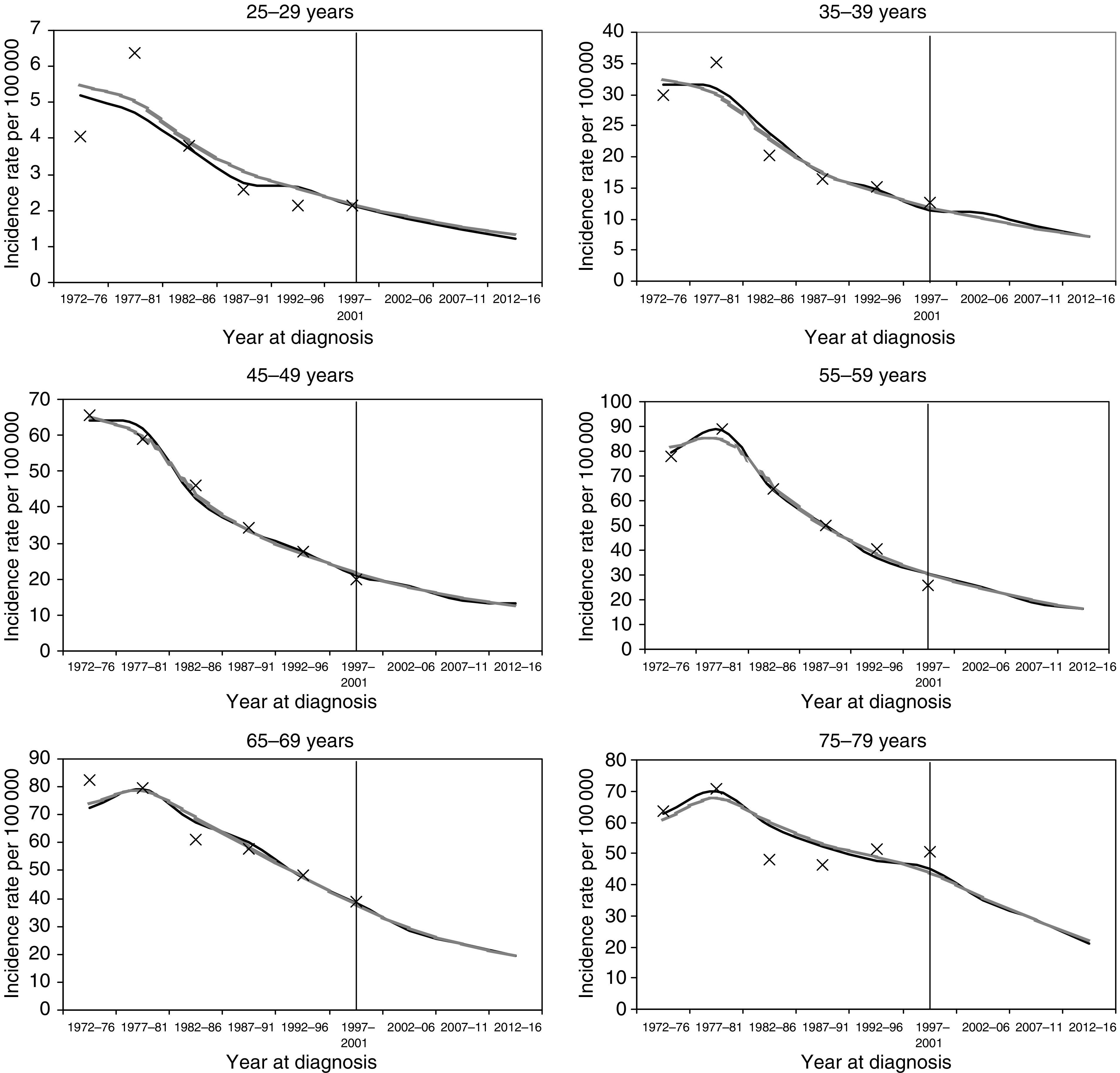
Observed compared to maximum likelihood (solid lines) and Bayesian posterior estimates (shaded lines) of fitted rates (1972–76 through 1997–2001) and empirical projections (2002–06 through 2012–16) of incidence by alternate 5-year age groups.

Figure 1 shows observed compared to maximum likelihood and Bayesian posterior estimates of the fitted rates (1972–76 through 1997–2001) and empirical projections (2002–06 through 2012–16) of incidence by alternate 5-year age groups in different panels. Visual inspection confirms that both model fits were generally good. The graphs suggest an overall decreasing trend, which was projected to continue in future periods.

### Incidence and mortality projections

**Figure 2:**
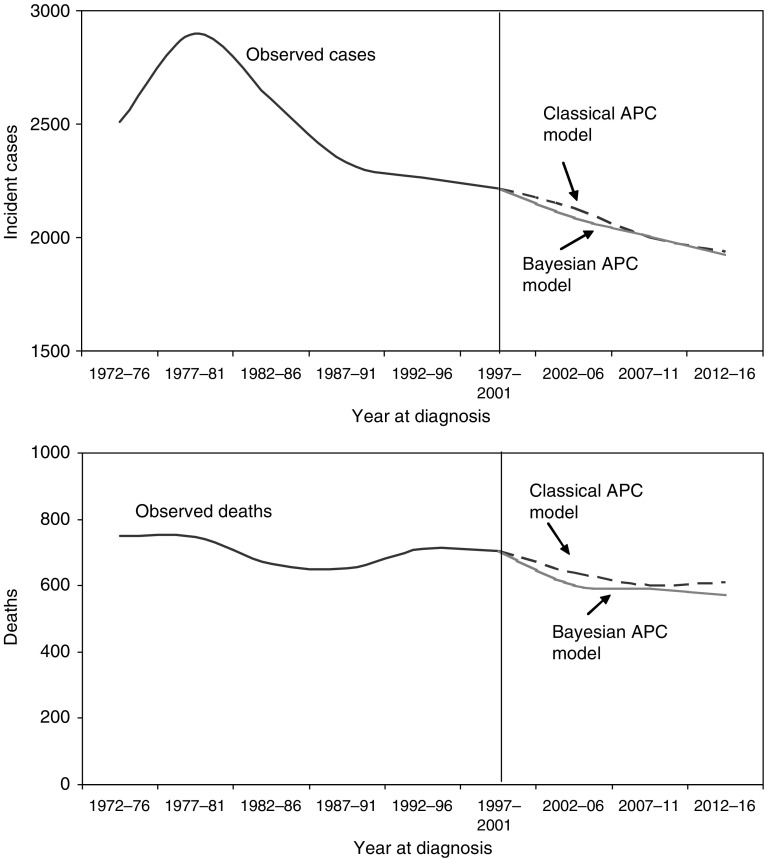
Observed and predicted cervical cancer incident cases and deaths from 1972 to 2016 with continuation of the *status quo* of opportunistic screening.

Figure 2 illustrates the number of fitted and projected cervical cancer cases and deaths from the maximum likelihood and Bayesian models under the base case scenario of opportunistic screening. The maximum likelihood model predicted a net of 51 more cervical cancer incident cases and 91 more death cases than the Bayesian methodology over the 15 years from 2002 to 2016. The maximum likelihood and Bayesian estimates were well within the 90% credible intervals and 95% confidence limits of each other, respectively (data not shown).

**Figure 3:**
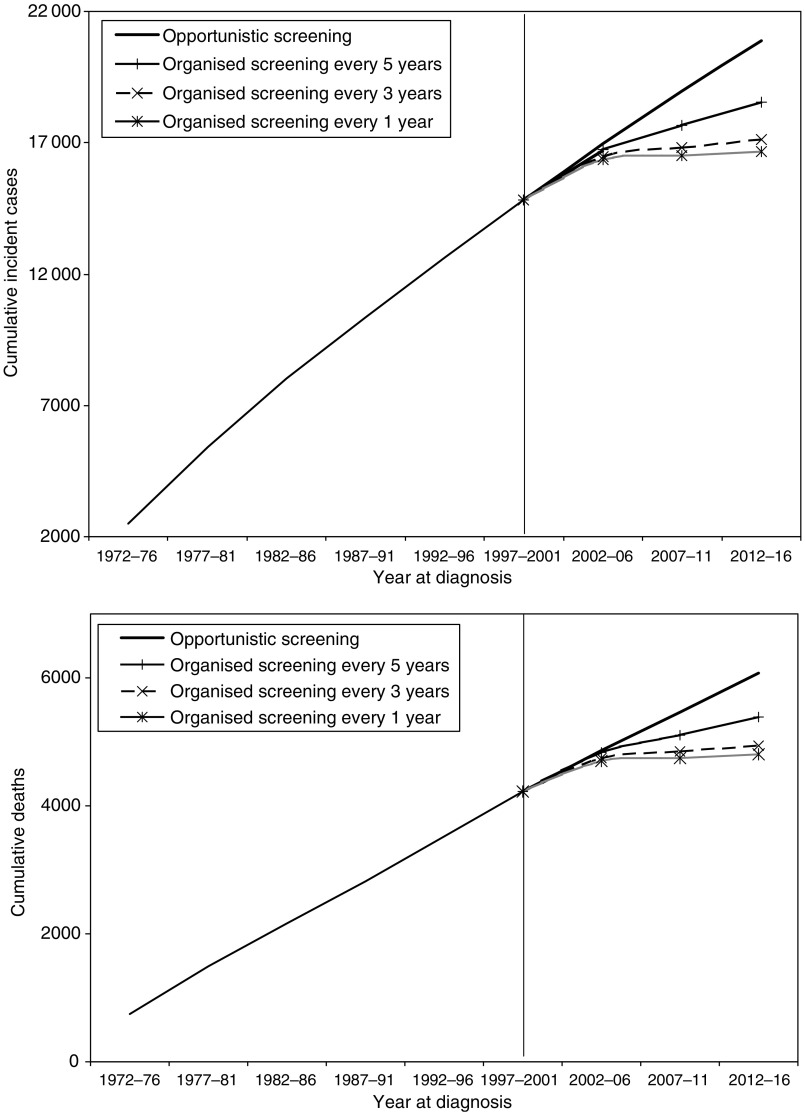
Cumulative incident cases and deaths from 1972 to 2016 under different screening scenarios by the maximum likelihood model.

Figure 3 shows the cumulative number of cervical cancer cases and deaths under the different screening policies. By the maximum likelihood model, 15-year projections to 2016 estimated that if all women were screened every 1, 3 and 5 years compared to the *status quo* of opportunistic screening, the incremental cumulative number of cases prevented (YLS) from 2002 to 2016 inclusive would be 4226 (33 000), 3778 (29 500) and 2334 (18 200), representing 70, 62 and 38% reductions, respectively. These cumulative YLS estimates correspond to those presented in Table 2, assuming 100% coverage. Table 2 also presents results under different screening coverage/uptake assumptions, namely 75 and 50%. In the sensitivity analysis where we varied future period and cohort effects by ±5% over the base case, the ranges (in parentheses) for the point estimates in Table 2 show that in the extreme cases where we assumed a −5% (+5%) change in both period and cohort effects in the same direction, the projected number of deaths would be adjusted by −17.2% (+25.0%). Table A of the appendix (online) presents the corresponding percentage change over the base case estimates for other scenarios. The graphs for the Bayesian model are almost identical to the maximum likelihood model and can be found in Figure C of the appendix (online) and the numerical estimates are included in Table 2.

## DISCUSSION

Analysis of secular trends in cancer epidemiology is important to the assessment of public health control policy. Cervical cancer mortality projections based on APC trends presented herewith suggest that the introduction of effective population screening could potentially prevent a continuing epidemic that would have culminated in about 4000 cancer deaths and 30 000 years of life by 2016, incremental to the current *status quo* of haphazard screening.

The direction and magnitude of our models and estimates are generally in line with other similar projection exercises reported in the literature. For instance, in England and Wales, Peto *et al* (2004) estimated that the National Health Service Cervical Screening Program, built on a history of opportunistic screening and which increased the smear coverage rate to 80%, was responsible for reducing cervical cancer incidence by 42% from the launch of the programme in 1988 up to 2000. In comparison, our model predicted that with organised screening aiming at 75% coverage, screening every 3 and 5 years would result in a comparable cancer incidence reduction of 44 and 39%, respectively, over the subsequent 15 years.

The present estimates are also comparable when benchmarked against historical observations. For example, in Sweden, organised screening was implemented in the mid-1960s and since then, there had been a steady decline in cervical cancer incidence of about 60% during 1959–93 (Dillner, 2000). Similarly, Finland's incidence dropped by 70–80% over the same period (Anttila and Nieminen, 2000). Based partly on Europe's experience, IARC (2005) estimated in a recent report that incidence reduction attributable to high-quality organised programme with virtually complete population coverage can be as high as 80%. Our model, assuming 100% coverage, predicted that new cancer cases could potentially decrease by 70% with screening every year and by 60% with screening every 3 years compared to the *status quo* of opportunistic testing.

In this projection exercise, we assumed that all women would derive the same level of benefit from cytologic screening irrespective of age and other risk characteristics in adopting the IARC figures (IARC Working Group, 1986). Our methods were predicated on the local screening programme achieving the same level of sustained effectiveness as that demonstrated in the IARC study starting from 2007. Nevertheless, we varied the population coverage proportions to demonstrate how the projected figures would change depending on screening uptake as per Table 2.

We did not model mortality trends directly because survival statistics are influenced by changes in treatment protocols and care patterns in addition to screening. Bonneux (2004) highlighted two common pitfalls of mortality models concerning the high level of uncertainty in estimating deaths averted from screening alone and a tacit assumption that cervical cancer risk in the future is constant by disregarding secular trends of incidence. In contrast, by focusing on incidence reduction through screening and extrapolating cancer deaths averted as a function of changes in incidence rates only, we were able to exclude the confounding effects of improved treatment and changing care practice over time. However, there is an important potential caveat associated with this approach concerning the underestimation of the benefit of screening. Specifically, screening can reduce cervical cancer mortality in two ways: (1) incidence reduction through early detection and treatment of precancerous lesions (CIN 1–3) before progression to invasive disease and (2) stage shift at the time of diagnosis towards earlier stages of invasive cancer thereby potentially improving survival. It is unclear how much of the overall benefit of cytologic screening, in terms of lowered mortality, can be attributed to each of these two components. Nevertheless, current consensus suggests that the former effect predominates in terms of number of deaths averted or YLS, although the verdict remains to be confirmed for outcome indices taking into account quality of life, for example, quality-adjusted life-years (QALYs) saved. Implicit in our *M*/*I* ratio method, we disregarded the latter effect owing to a lack of stage-specific incidence data in the local disease registry, and that all invasive tumours have been recorded as cancer cases irrespective of stage. Therefore, we could have potentially underestimated the magnitude of mortality reduction as a result of screening.

From the methodological viewpoint, we built on previous work (Dyba and Hakulinen, 2000; Bashir and Esteve, 2001; Møller *et al*, 2003) on comparing different maximum likelihood APC modelling techniques by extending the methodology to a Bayesian approach. We adopted the second-order autoregressive Poisson model, recognised as likely the most reliable maximum likelihood technique as per Bashir and Esteve (2001) and Dyba and Hakulinen (2000), as the maximum likelihood comparator to the Bayesian method and found that both analytic approaches yielded very similar estimates, thus lending added credibility to the projected estimates.

In terms of policy implementation, we note a practical caveat concerning the predictive relevance of our findings vis-à-vis the organised screening programme launched in Hong Kong in 2004 (http://www.cervicalscreening.gov.hk/). Currently, as a result of resource constraints, this government-operated programme mainly provides a prospective record and recall function for those who have ever been screened. The programme encourages women to undergo regular cytologic examination through social marketing campaigns for the general public and via primary care and women's health providers to individuals on an opportunistic basis. For those women who decide to get screened, they can seek to be tested at public or private care providers on a full fee-for-service basis using either traditional pap smear or liquid-based method with/without human papilloma virus (HPV) testing. These providers are then encouraged to enter the screened woman's details into a centralised database for subsequent automatic recall (every 3 years) and archiving of test results. Therefore, without explicitly anchoring the programme with proactive, personalised invitation to be screened (initial ‘call’ function) and direct provision of pap testing at dedicated facilities for a reasonable fee (as opposed to full market rates), it is a suboptimal arrangement by Hakama and *et al*'s (1985) definition of an ideal programme. Nevertheless, referral for colpolscopy and subsequent management in the case of invasive disease are available in both the public and private sectors, where the former essentially provides universal access to all services with very minimal co-payments (amounting to an all-inclusive per diem charge of less than £7 or $13) at the point of care. Indeed, the public sector provides 95% of total bed-days for all in-patient care locally. As at March 2005 after 12 months in operation, just under 120 000 (4.8%) women out of a potentially eligible female population of 2.5 million aged 21–69 years have been registered, even though we know that between 42 and 60% of local women reported being ever screened in the past 5 years. It remains to be seen whether the projected benefits of organised screening can be realised, perhaps with modification and enhancements subsequently to the present programme arrangements.

Lastly, our models did not take into account scientific advances in cancer biology and related research. There has been unprecedented progress in our understanding of the origin and pathogenesis of cervical neoplasia in the past decade. With the prospect of primary prevention of cervical cancer by a prophylactic HPV vaccine and/or secondary/tertiary prevention by a therapeutic vaccine at the stages of *in situ* or invasive disease on the horizon (Rohan *et al*, 2003), recombinant DNA technologies may render the prevention and eradication of the vast majority of cervical cancer cases a real possibility.

## Figures and Tables

**Table 1:** Summary statistics comparing goodness-of-fit for different maximum likelihood models

**Model**	**Degrees of freedom**	**Deviance**	***P*-value**
Age	60	1805.2	
Age–drift	59	270.9	
Age–period	55	214.6	<0.001
Age–cohort	44	111.0	<0.001
Age–period–cohort	40	68.5	

**Table 2:** Years of life saved derived from both maximum likelihood and Bayesian models by adopting a population-based cytologic screening call–recall programme *vs* the *status quo* of opportunistic screening

	**Every 5 years**	**Every 3 years**	**Every 1 year**
*Maximum likelihood model*			
100% coverage	18 200 (15 100, 22 700)	29 500 (24 400, 36 800)	33 000 (27 300, 41 200)
75% coverage	10 900 (9000, 13 600)	18 700 (15 500, 23 400)	20 800 (17 200, 26 000)
50% coverage	3900 (3200, 4900)	8900 (7400, 11 200)	10 300 (8600, 12 900)
			
*Bayesian model*			
100% coverage	17 900 (14 900, 22 400)	29 300 (24 200, 36 600)	32 800 (27 200, 41 000)
75% coverage	11 000 (9100, 13 700)	18 800 (15 600, 23 500)	20 900 (17 300, 26 100)
50% coverage	4000 (3300, 5000)	9000 (7500, 11 300)	10 100 (8400, 12 600)

Numbers are rounded to the nearest hundred. Ranges (in parentheses) around the point estimates were calculated by varying the percentage change in future period and cohort effects per 5-year time period from −5 to 5% over the base case values. A −5% change in both period and cohort effects yielded an overall percentage change of −17.2%, while a +5% change in both period and cohort effects resulted in an overall percentage change of +25.0% over the 15-year projected period.
