# Supplementary material for: Modelling the impact of population-based cytologic screening on cervical cancer incidence and mortality in Hong Kong: an age–period–cohort approach
Source: Br J Cancer. 2005 Oct 4;93(9):1077–83. doi: 10.1038/sj.bjc.6602805 (PMC2361667; doi:10.1038/sj.bjc.6602805)
Supplement: Appendix (online only) [file 93-6602805x1.doc]

**APPENDIX (online only)**

**Figure A** Bayesian convergence diagnostic plots for selected parameters of age, period, cohort and incidence rate

| Age group (50–54 years) |
| --- |
| 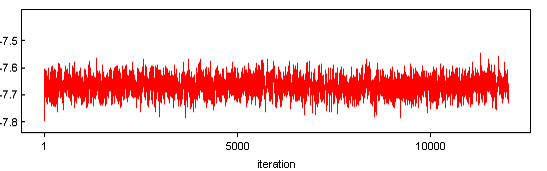 |
| Year at diagnosis (2002–06) |
| 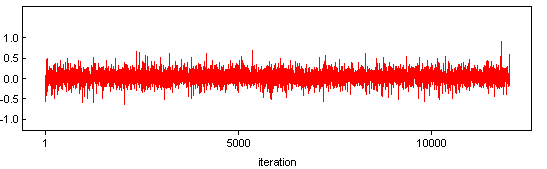 |
| Birth cohort (1942) |
| 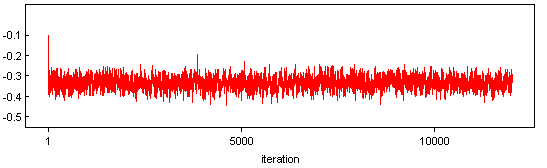 |
|  |

**Figure B** Autocorrelation plots for 1,000 samples of selected age, period and cohort parameters

| **Age group** |  |  |
| --- | --- | --- |
| 25–29 years | 45–49 years | 75–79 years |
| **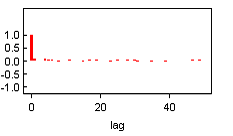** | **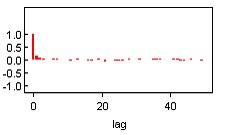** | **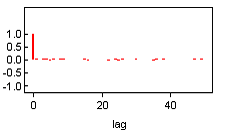** |
| **Year at diagnosis** |  |  |
| 1972–76 | 1992–96 | 2012–16 |
| 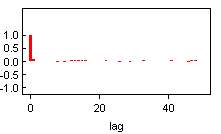 | 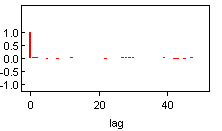 | 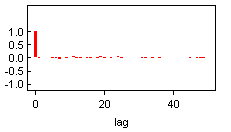 |
| **Birth cohort** |  |  |
| 1892 | 1937 | 1987 |
| 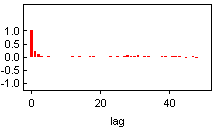 | 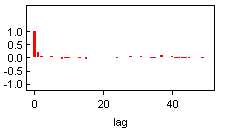 | 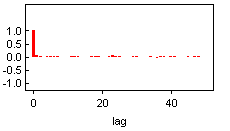 |

**Figure C Cumulative incident cases and deaths from 1972 to 2016 under different screening scenarios by the Bayesian model**

|  |
| --- |
|  |

**Table A** Overall percentage change in mortality over the 15-year projected period compared with the base case
